# Retraction: By downregulating TIAM1 expression, microRNA-329 suppresses gastric cancer invasion and growth

**DOI:** 10.18632/oncotarget.28830

**Published:** 2026-04-17

**Authors:** Zheng Li, Xin Yu, Yang Wang, Jianxiong Shen, William Ka Kei Wu, Jinqian Liang, Fan Feng

**Affiliations:** ^1^Department of Orthopaedic Surgery, Peking Union Medical College Hospital, Chinese Academy of Medical Sciences and Peking Union Medical College, Beijing, China; ^2^Department of Abdominal Surgery, Cancer Institute and Cancer Hospital, Peking Union Medical College and Chinese Academy of Medical Sciences, Beijing, China; ^3^Department of Anaesthesia and Intensive Care, The Chinese University of Hong Kong, Hong Kong, China

**This article has been retracted:** Oncotarget has completed its investigation of this paper. It was determined that the article contains multiple instances of image duplication and overlap, both internal to the paper and external with other published works. Specifically, we identified the following issues:

Internal Duplications and Overlaps:

- Figure 3 (Transwell assay): panel 3A/migration-scramble was reused in 3C/invasion as control; panel 3A/migration-miR-329 inhibitor, overlaps with 3C/invasion-miR-329 inhibitor; panel 3B/migration-control overlaps with panels 3D/invasion-control and 3D/invasion-scrambled; and panel 3B/migration-miR-329 inhibitor overlaps with panel 3D/invasion miR-329 inhibitor.
- Figure 5D (Invasion): panel 5D/HGC-27 cells/control overlaps with 5D/HGC-27 cells/TIAM1+miR-329, and panel 5D/MGC-803 cells/control overlaps with 5D/MGC-803 cells/TIAM1+miR-329.

External Duplications and Overlaps:

- Image overlaps or duplications were found between the transwell assays in Figures 3A, 3D, and 5D and panels 2C and 5D in reference [1].
- Panel 5D overlaps with panel 5C in reference [2].
- Panels in Figures 3A and 3C overlap with Figures 6M and 3J from reference [3], respectively.
- The Western blot for GAPDH in Figure 4D was found in Figures 4A of [4], 4A of [5], 7E of [6], 5B of [7], and 5B of [8]. The GAPDH in Figure 6B was found in reference [9].

Additionally, the corresponding author, Dr. Shen Jianxiong, requested the removal of his name from this publication. He stated that, as a spine surgeon, he has never conducted gastric cancer research and had no access to the study’s data.

This article was identified as part of a series of submissions involving concerns regarding authorship and similarities across multiple papers. These issues call into question the validity of the reported results. Consequently, the editorial decision has been made to retract this article. The authors have been notified of this decision, but did not reply.

Original article: Oncotarget. 2015; 6:17559–17569. 17559-17569. https://doi.org/10.18632/oncotarget.2755
